# Myostatin-1 Inhibits Cell Proliferation by Inhibiting the mTOR Signal Pathway and MRFs, and Activating the Ubiquitin-Proteasomal System in Skeletal Muscle Cells of Japanese Flounder *Paralichthys olivaceus*

**DOI:** 10.3390/cells9112376

**Published:** 2020-10-29

**Authors:** Jiahuan Liu, Mingzhu Pan, Dong Huang, Yanlin Guo, Mengxi Yang, Wenbing Zhang, Kangsen Mai

**Affiliations:** The Key Laboratory of Aquaculture Nutrition and Feeds (Ministry of Agriculture and Rural Affairs), the Key Laboratory of Mariculture (Ministry of Education), Ocean University of China, Qingdao 266003, China; liujiahuan@stu.ouc.edu.cn (J.L.); pmz@stu.ouc.edu.cn (M.P.); huangdong@stu.ouc.edu.cn (D.H.); guoyanlin@stu.ouc.edu.cn (Y.G.); ymx@stu.ouc.edu.cn (M.Y.); kmai@ouc.edu.cn (K.M.)

**Keywords:** myostatin, muscle cell, mTOR, Forkhead box O1, ubiquitin-proteasomal system

## Abstract

Myostatin (MSTN) is a negative regulator of skeletal muscle growth and development. The mechanisms of fish MSTN involved in muscle growth are not fully understood. In the present study, knockdown and overexpression of *mstn-1* was performed in cultured Japanese flounder muscle cells to investigate the molecular function and the underlying mechanism of fish MSTN-1. Results showed that *mstn-1* knockdown significantly induced cell proliferation and the mRNA expression of myogenic regulatory factors (MRFs), while overexpression of *mstn-1* led to a significant decrease of cell proliferation and a suppression of the MRFs mRNA expression. The overexpression of *mstn-1* also significantly increased the mRNA expression of ubiquitin–proteasomal pathway of proteolysis genes including muscle RING-finger protein 1 (*murf-1*) by 204.1% (*p* = 0.024) and muscle atrophy F-box protein (*mafbx*) by 165.7% (*p* = 0.011). However, *mystn-1* overexpression inhibited the activation of mTOR signal pathway and the AKT/FoxO1 pathway through decreasing phosphorylation of AKT at Ser 473 by 56.0% (*p* = 0.001). Meanwhile, *mystn-1* overexpression increased the dephosphorylation and nuclear localization of FoxO1 by 394.9% (*p* = 0.005). These results demonstrate that *mstn-1* in Japanese flounder has the effects of inhibiting cell proliferation and growth, and the mTOR and AKT/FoxO1 pathways participated in these biological effects.

## 1. Introduction

Myostatin (MSTN), which is also known as growth and differentiation factor 8 (GDF8), is a member of transforming growth factor-β (TGF-β) superfamily. The most important function of MSTN is regulating skeletal muscle growth [1,2,3]. Research in mice found that the deletion of MSTN induces the increase in skeletal muscle mass due to both muscle hypertrophy and hyperplasia [1,4,5], while overexpression of MSTN can cause dramatic atrophy of skeletal muscle [5,6].

Two distinct MSTN clades named MSTN-1 and MSTN-2 can be found in some fish genomes due to an early genome duplication in the teleost fish lineage [7,8]. Another duplication occurred in each clade within the salmonids during the evolvement of this lineage, as a result, four MSTN genes (MSTN-1a, MSTN-1b, MSTN-2a, and MSTN-2b paralogs) were located in their genome [9,10]. The expression pattern of *mstn* in fish is distinctive [11,12,13]. In fish, *mstn* mRNA can be detected in various tissues including skeletal muscle, eyes, ovary, brain, and kidney [13,14,15], whereas in mammals, it is expressed specifically in skeletal muscle. Studies have demonstrated that the functions of MSTN in fish and mammals were not completely remained conserved during evolution [6,16,17]. Since skeletal muscle is the main component and edible part of fish. The investigation of possible mechanism, which controls muscle growth, may result a promotion of production in aquaculture industry. Therefore, the function of MSTN and its molecular mechanism on muscle growth in fish are worth studying.

Studies have indicated that MSTN negatively regulates skeletal muscle growth in some fish like in mammalian species. Knockdown of *mstn-1* gene in zebrafish (*Danio rerio*) upregulated the myogenic regulatory factors and therefore increased the size of somites [18]. Giant phenotype and increase in skeletal muscle mass were found in many studies of different fish species, including zebrafish [19,20,21], channel catfish (*Ictalurus punctatus*) [22], red sea bream (*Pagrus major*) [23], Japanese flounder (*Paralichthys olivaceus*) [24], and yellow catfish (*Pelteobagrus fulvidraco*) [25], when carrying MSTN knockdown genotype. Xu et al. (2003) observed a significant increase in fiber number in transgenic zebrafish that overexpress MSTN prodomain (a binding protein of MSTN) [26]. Treatment with goldfish MSTN receptor (activin type IIB receptor), which was assumed to disrupt the function of MSTN, stimulated the growth of goldfish (*Carassius auratus*), African catfish (*Clarias gariepinus*), and tilapia (*Oreochromis aureus*), as a result of increased muscle weight [27]. In rainbow trout (*Oncorhynchus mykiss*), overexpression of follistatin (an inhibitor of MSTN) induced increase in both size and number of muscle cells [28].

However, the underlying mechanisms of MSTN involved in muscle growth of fish are not fully understood. In the study of rainbow trout, human recombinant MSTN treatment inhibited the activation of the growth-promoting mTOR signaling pathway. At the same time, it also induced the myotubes atrophy through stimulating the catabolic route in rainbow trout myotubes [29]. This study suggests that MSTN in fish may act through the mTOR signal pathway and proteolytic signaling pathway.

Japanese flounder (*Paralichthys olivaceus*) is one of the most commercially important aquaculture fish species in East Asia. In 2008, MSTN-1 gene was isolated and characterized in Japanese flounder [13]. Lee et al. (2010) found that treating with recombinant MSTN-1 prodomains of Japanese flounder promoted the growth of rainbow trout [30]. Another study showed Japanese flounder exhibited enhanced muscle mass with muscle hyperplasia by CRISPR/Cas9-mediated MSTN-1 disruption [24]. These researches illustrated the potential molecular biological function of Japanese flounder MSTN-1. As research on the MSTN pathway in fish with regards to using cell-based systems is limited, the better understanding of Japanese flounder endogenous myostatin-1 will provide a perspective to control muscle mass and quality in aquaculture by controlling MSTN activity. In the present study, an in vitro system of Japanese flounder muscle cells culture was used to partly reflect the molecular function and the underlying mechanism of fish MSTN-1.

## 2. Materials and Methods

### 2.1. Ethical Statement

The present study was performed in strict accordance with the recommendations in the Guide for the Use of Experimental Animals of Ocean University of China. The protocols for animal care and handing used in this study were approved by the Institutional Animal Care and Use Committee of Ocean University of China.

### 2.2. Animals

Japanese flounder with weight ranging from 8 to 10 g were purchased from a commercial fish farm in Haiyang city (Shandong, China). After disinfection, fish were kept in 0.4 m^3^ tanks with a circulating water system at 23 °C.

### 2.3. Primary Cell Cultures

The muscle cells of Japanese flounder were isolated according to the protocols described by Vegusdal et al. (2004) [31] and Jiménez-Amilburu et al. (2013) [32] with some modifications. The fish were killed by a blow to the head and then were immersed in 70% ethanol for 1 min to sterilize the external surfaces. White epaxial muscle was excised under sterile conditions and washed by phosphate buffer solution (PBS) (HyClone, Logan, UT, USA), then the muscle was transferred into Dulbecco’s Modified Eagle Medium/Nutrient Mixture F-12 (DMEM/F12) (HyClone, Logan, UT, USA) containing antibiotics (Penicillin-Streptomycin, 100 U/mL) (HyClone, Logan, UT, USA). The tissue was minced and the fragments were centrifuged at 300× *g* for 5 min. After centrifugation, the fragments were washed twice in DMEM/F12 containing antibiotics (Penicillin-Streptomycin, 100 U/mL) to eliminate erythrocyte. Type II collagenase (0.2%) (MP Biomedicals, Solon, OH, USA) was used to digest the tissue fragments for 90 min at 23 °C with gentle shaking. The suspension was centrifuged at 300× *g* for 5 min and the pellet was then resuspended in a trypsin solution (0.1% final concentration in DMEM/F12) (HyClone, Logan, UT, USA). The suspension containing fragments was digested for 20 min at 23 °C with gentle agitation before centrifugation at 300× *g* for 1 min. The supernatant was collected in 2 volumes of cold DMEM/F12 containing fetal bovine serum (FBS) (Bioind, Kibbuiz, Israel) to terminate trypsin digestion. The tissue fragments were subjected to a second trypsin digestion and centrifugation under the same conditions, and the supernatant was diluted in 2 volumes of DMEM/F12 containing FBS. The two supernatants were amalgamated and centrifuged at 300× *g* for 15 min. The resulting pellet was resuspended in complete medium (DMEM/F12 supplemented with 10% FBS, 2 mM L-glutamine, and antibiotics) and filtered through a 40-μm nylon cell strainer. The cells were diluted in complete medium and plated on 6-well plates (Corning, Lowell, MA, USA) at 1 × 10^6^ cells per mL medium. Cells were incubated at 23 °C without CO_2_. After the overnight adhesion, the cells were washed with medium, and the medium was changed every 2 days. The morphology was observed regularly to control the state of the cells. For the subsequent research, muscle cells (80–90% confluency) at day 4 were used.

### 2.4. mRNA-Expression of Muscle-Specific Proteins and Gene Expression

RNA from cells was extracted by TRIzol (Invitrogen, Carlsbad, CA, USA) and quantified on a spectrophotometer (NanoDrop 2000, Thermo Fisher Scientific, Wilmington, DE, USA). Reverse transcription was performed using PrimeScript^®^ RT Reagent Kit with gDNA Eraser (Perfect Real Time, Takara, Shiga, Japan). The quantity of cDNA for each transcript was analyzed on the ABI 7500 system (Applied Biosystems, Foster, CA, USA) using TB Green Fast qPCR Mix (Takara, Shiga, Japan). Relative quantifies of target genes were calculated by the ΔΔCt method using *β-actin* gene expression as reference. All the primers used in present study are listed in Table 1.

The mRNA-expression of muscle-specific proteins were tested using reverse-transcriptase PCR (RT-PCR) analyses on a Biometra TRIO-Thermoblock 48 (Biometra GmbH, GoÈttingen, Germany). The amplified products were detected and visualized by agarose gel electrophoresis followed by GelRed (Invitrogen, Carlsbad, CA, USA) staining. The primers used for the RT-PCR analysis are listed in Table 2.

### 2.5. In Vitro Screening of siRNAs and mstn-1 Interfering

Six siRNA duplexes (siRNA-162, siRNA-315, siRNA-578, siRNA-677, siRNA-912, and siRNA-1070, respectively) targeting different encoding regions of *mstn-1* and a silence negative control siRNA (si*mstn-1*-NC) were designed and synthesized by Sangon (Sangon Biotech, Shanghai, China) (Supplementary Table S1).

Muscle cells were seeded in 6-well plates (Corning, Lowell, MA, USA) at a density of 1.0 × 10^6^ cells/well and incubated at 23 °C. After 96 h, the cells at 80–90% confluency were transfected with siRNAs. For transfection, 5 μg of siRNA and 3.75 μL of Lipofectamine 3000 reagent (Invitrogen, Carlsbad, CA, USA) were used in each well according to the manufacturer’s protocol. Scrambled shRNA (si*mstn-1*-NC) served as a negative control for the experiment. No siRNA but an equal amount of PBS (Sangon Biotech, Shanghai, China) was added as a control group. Cells were harvested after 24 h to determine transfection efficiency by qPCR. The transfection experiments were performed in triplicate. Experiments would be subsequently performed to assess the silence effects only if the transfection effects were >60%.

### 2.6. Overexpression of mstn-1 in Japanese Flounder Muscle Cells

#### 2.6.1. Plasmid Construction

The *mstn-1* gene coding sequence (CDS) was amplified using specific primers consisting of the forward primer *mstn-1*-cds-F: 5’-ATGCATCTGTCTCACATTGTGCTCT-3’ and the reverse primer *mstn-1*-cds-R: 5’-AGAGCACCCGCAACGGTCCA-3’. The PCR product with a length of 1131 bp was examined on 1% agarose gel and purified using the SanPrep Column DNA Gel Extraction Kit (Sangon Biotech, Shanghai, China). The purified DNA fragments were inserted into the pEASY-T1 Simple Cloning Vector (TransGen Biotech, Beijing, China) and used for transformation of Trans1-T1 Phage Resistant Chemically Competent Cell (TransGen Biotech, Beijing, China). Clones with inserts were sequenced in both forward and reverse directions using the universal M13 primers (Huada Genomics Co. Ltd., Beijing, China), and the constructed vector was confirmed by DNA sequencing. The templates *mstn-1* was PCR amplified using primers with homology arms to BamHI region in pcDNA3.1-EGFP consisting of the forward primer *mstn-1*-hr-F: 5’- cttggtaccgagctcggatccATGCATCTGTCTCACATTGTGCTC-3’ and the reverse primer *mstn-1*-hr-R: 5’-atggtggcgaccggtggatccAGAGCACCCGCAACGGTC-3’. The resulting amplicons were purified by gel electrophoresis and extracted using the SanPrep Column DNA Gel Extraction Kit. The pcDNA3.1-EGFP plasmid (Biofeng, Shanghai, China) was digested with enzyme BamHI. The purified resulting amplicons was then ligated into the pcDNA3.1-EGFP plasmid using T4 DNA ligase (TransGen Biotech, Beijing, China). The plasmid constructed was called pcDNA3.1-MSTN-1-EGFP. The construct was confirmed by DNA sequencing. The pcDNA3.1-MSTN-1-EGFP plasmid was then transformed into the *Escherichia coli* strain DH5α. After shaking the flask culture overnight, adequate plasmid for transfection was collected from the *E. coli* strain DH5α using a EasyPure HiPure Plasmid MaxiPrep Kit (TransGen Biotech, Beijing, China).

#### 2.6.2. Overexpression of mstn-1

Muscle cells were seeded in 6-well plates (Corning, Lowell, MA, USA) at a density of 1.0 × 10^6^ cells/well and incubated at 23 °C. After 96 h, the cells at 80–90% confluency were transfected with pcDNA3.1-MSTN-1-EGFP using Lipofectamine 3000 (Invitrogen, Carlsbad, CA, USA) according to manufacturer’s instruction. For each well, 2.5 μg of plasmid was used. The pcDNA3.1-EGFP plasmid served as a negative control for the experiment. No plasmid but an equal volume of PBS (Sangon Biotech, Shanghai, China) was added as a control group. After 24 h, transfection efficiency was determined using Fluorescence microscope (Echo Laboratories, San Diego, CA, USA) and qPCR. EGFP-positive cells were calculated using Image-Pro Plus 6.0 software (Media Cybernetics, Silver Spring, MD, USA). The transfection experiments were performed in triplicate. The expressions of muscle growth-related genes and proteolysis-related genes were also detected by real-time RT-PCR as described in Section 2.4.

### 2.7. Proliferation Assay

Cell proliferation assays were conducted in 48-well plates. After a 24 h attachment period, the cells were transfected with siRNA-578 and pcDNA3.1-MSTN-1-EGFP plasmid, respectively. The si*mstn-1*-NC and pcDNA3.1-EGFP plasmid were used as negative controls, respectively. The plates were then incubated at 23 °C for 24, 48, 72, and 96 h. After the incubation period, the cell proliferation assay was performed by adding 20 μL of Cell-Counting Kit-8 (CCK-8) reagents (Sigma, St. Louis, MO, USA) to each well of the plate for 4 h. Finally, the absorbance at 450 nm was measured using a spectrophotometer (UV-2401PC, Shimadzu, Kyoto, Japan).

### 2.8. Western Blot Analysis

Forty-eight hours after the *mstn-1* overexpression treatment, the medium was removed and cells were washed three times with 2 mL PBS per well and lysed in 200 μL radioimmunoprecipitation lysis buffer (Solarbio Science and Technology Co., Ltd., Beijing, China) supplemented with protease and phosphatase inhibiter cocktail (Roche, Indianapolis, IN, USA) at 0 °C for 30 min. Homogenates were centrifuged at 12,000× *g* for 10 min at 4 °C, and the protein concentration in the supernatant was determined using a Bicinchoninic Acid Protein Assay Kit (Beyotime Institute of Biotechnology, Nanjing, China). Nuclear protein was extracted using NE-PER Nuclear and Cytoplasmic Extraction Reagents Kit (Thermo Fisher Scientific, Waltham, MA, USA) according to the manufacturer’s instruction. Equal amounts of protein were separated by sodium dodecyl sulfate-polyacrylamide gels (SDS-PAGE). Gels were cut according to molecular weight and transferred to 0.45 μm PVDF membrane (Millipore, Billerica, MA, USA). Different target proteins were tested in independent membrane, respectively. Incubation with the primary antibody was performed overnight at 4 °C. The primary antibodies used were MSTN (dilution 1:1000, R&D Systems, Minneapolis, MN, USA, cat. No. MAB788), phospho-AKT (ser473) (dilution 1:1000, Wanleibio, Shenyang, China, cat. No. WLP001), phospho-AKT (Thr308) (dilution 1:500, Affinity, Cincinnati, OH, USA, cat. No. AF3262), AKT (dilution 1:1000, Protein Tech, Rosemont, IL, USA, Cat. No. 60203-2-lg), phosphor-Forkhead box O1 (FoxO1) (Thr24) (dilution 1:1000, Cell Signaling Technologies, Danvers, MA, USA, Cat. No.9464), FoxO1 (dilution 1:1000, Beyotime Institute of Biotechnology, Nanjing, China, cat. No. AF1600), phospho-mTOR (Ser2448) (dilution 1:1000, Cell Signaling Technology Inc., Danvers, MA, USA, Cat. No. 2971), mTOR (dilution 1:1000, Cell Signaling Technology Inc., Danvers, MA, USA, Cat. No. 2972), phospho-S6 (Ser235/236) (dilution 1:2000, Cell Signaling Technology Inc., Danvers, MA, USA, Cat. No. 4858), S6 (dilution 1:1000, Cell Signaling Technology Inc., Danvers, MA, USA, Cat. No. 2217), Lamin B1 (dilution 1:500, Wanleibio, Shenyang, China, cat. No. WL01775), and β-actin (dilution 1:5000, Bioss Antibodies, Woburn, MA, USA, Cat. No. bs-0061R). After the incubation, the membrane was washed with TBST and incubated with secondary antibody (HRP-labeled goat anti-Rabbit lgG) (Beyotime Institute of Biotechnology, Nanjing, China) at 1:5000 dilution for 1 h at room temperature. After that, the membrane was developed with Beyo ECL Plus reagent (Beyotime Institute of Biotechnology, Nanjing, China) and exposed to the X-ray file. The band densities were quantified using ImageJ software (National Institutes of Health, Bethesda, MD, USA).

### 2.9. Statistical Analysis

All statistical analyses were conducted using software SPSS 22.0 (IBM Corp., Armonk, NY, USA). The silencing efficiency of the siRNAs was analyzed by one-way analysis of variance (ANOVA) followed by Tukey’s multiple range tests. Other statistical evaluations were analyzed by *t*-test compared with the control group. All data were expressed as means ± SE. Differences were considered significant when *p* < 0.05. 

## 3. Results

### 3.1. Culture and Proliferation of Muscle Cells In Vitro

**Figure 1:**
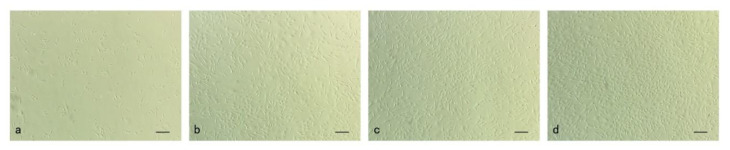
Micrographs of Japanese flounder muscle cells in primary culture; bar = 100 µm. (**a**) 1 day after seeding, sticking to the wall and extension of muscle cells were observed; (**b**) 3 day after seeding, completely stretching and proliferation were observed; (**c**) 4 day after seeding, the cells reached a fusion rate of 80–90%; (**d**) 5 day after seeding, the cells overspread the culture bottle bottom.

One day after seeding, sticking to the wall and extension of muscle cells were observed (Figure 1a). On the third day in culture, completely stretching and proliferation were observed (Figure 1b). Four days after seeding, the cells reached a fusion rate of 80–90% (Figure 1c). Five days after seeding, the cells overspread the bottom of culture bottle, and the muscle cells elongated and started to fuse with other cells (Figure 1d).

### 3.2. Expression of mRNA of Muscle-Specific Proteins

**Figure 2:**
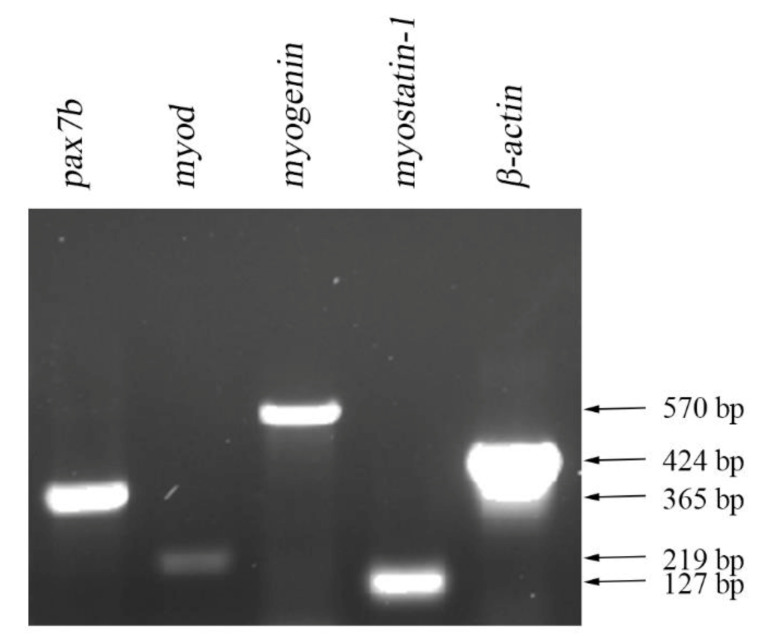
Expression of mRNA of myogenic regulatory proteins in primary cultured Japanese flounder muscle cells. The cDNA sequences of *pax7b*, *myod*, *myogenin,* and *myostatin-1* were amplified by reverse-transcriptase PCR from total RNA. *β-actin* was included as a positive control.

The mRNA of myogenic regulatory proteins in primary cultured Japanese flounder muscle cells were amplified by reverse-transcriptase PCR from total RNA. The mRNA of pax7b, myod, myogenin (myog), and myostatin-1 were all expressed after 7 day in culture (Figure 2). β-actin was included as a positive control.

### 3.3. Knockdown of mstn-1 Expression by mstn-1-siRNA in Primary Muscle Cells

**Figure 3:**
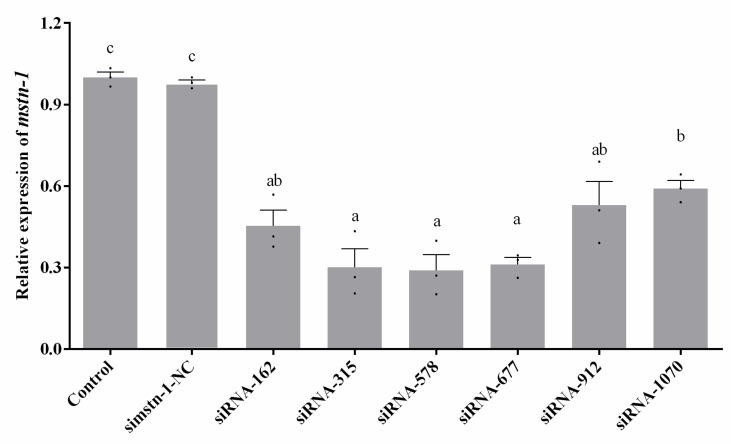
Relative level of *mstn-1* mRNA in primary cultured muscle cells treated with si*mstn-1*. Results are represented as mean ± SE (*n* = 3). Values with different letters mean significant differences (*p* < 0.05). Letter **a** and **c** represent the lowest value and the highest value respectively. Letter **b** represents the value significantly higher than **a** but significantly lower than **c**. Letter **ab** represents the value significantly lower than **c** but has no significant difference with **a** and **b**.

Six siRNAs and scrambled siRNA were evaluated for their *mstn-1* gene silencing efficiency in Japanese flounder muscle cells to screen the most effective siRNA duplex. The scrambled siRNA (si*mstn-1*-NC) did not affect the expression of *mstn-1* compared with the control group. All six siRNAs showed significant reductions (*p* < 0.05) in the level of *mstn-1* mRNA compared to the scrambled siRNA transfected cells (Figure 3). The siRNA-315, siRNA-578, and siRNA-677 were highest in silencing efficiency without significant difference among them (*p* > 0.05), while siRNA-578 had the highest knockdown efficiency in value (decreasing about 71%). The result indicated that siRNA-315, siRNA-578, and siRNA-677 were the most efficient duplexes for knocking down *mstn-1* expression in Japanese flounder muscle cells, and siRNA-578 duplex was used in the formal experiments of *mstn-1* interfering. *Myostatin-1* knockdown via si-*mstn-1* (siRNA-578) transfection significantly reduced the *mstn-1* mRNA level.

### 3.4. Relative Expression of Muscle Growth-Related Genes after Knockdown of mstn-1 in Muscle Cells of Japanese Flounder

**Figure 4:**
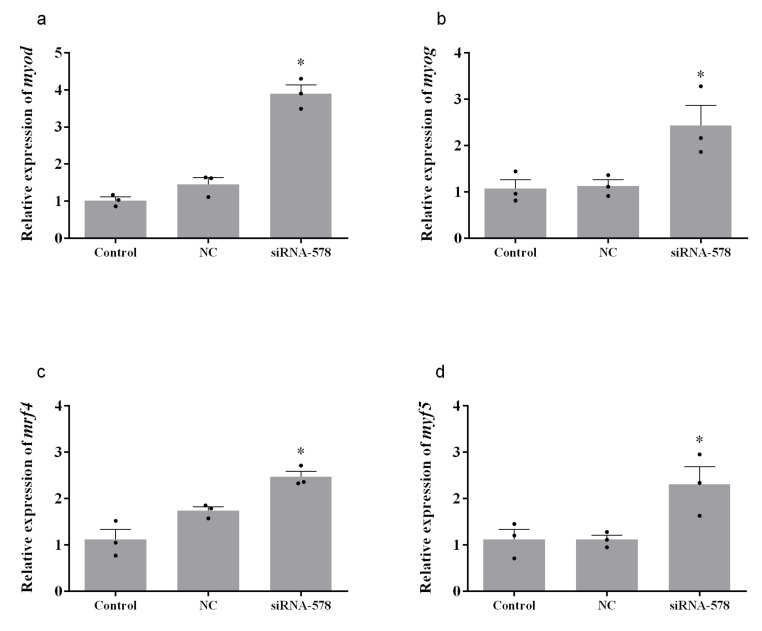
Relative expression of muscle growth-related genes in primary cultured muscle cells treated with si*mstn-1*. (**a**) Relative expression of *myod*. (**b**) Relative expression of *myog*. (**c**) Relative expression of *mrf4*. (**d**) Relative expression of *myf5*. Results are represented as mean ± SE (*n* = 3). * means significantly different compared with control (*p* < 0.05).

After 24 h transfection by *mstn-1*-siRNA, the mRNA levels of *myod*, *myog*, *mrf4,* and *myf5* significantly increased compared with the control group (*p* < 0.05), while si*mstn-1*-NC did not affect the expression of muscle growth-related genes (*p* > 0.05). The data are shown in Figure 4.

### 3.5. Transfection Efficiency, mstn-1 mRNA, and Protein Expression Analysis after mstn-1 Overexpression

**Figure 5:**
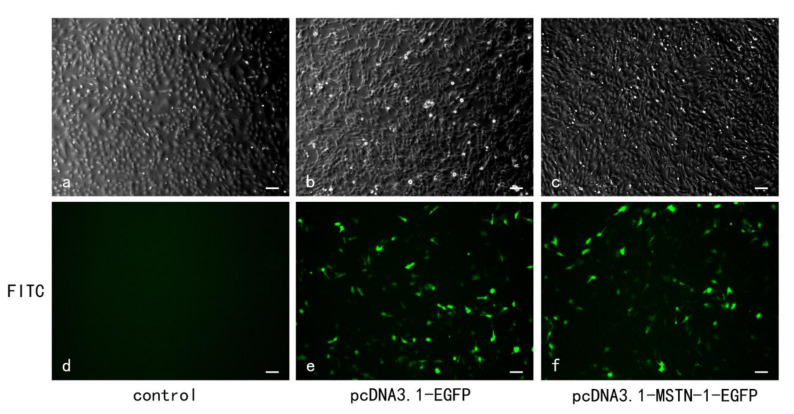
The Images of muscle cells after transfection at 24 h; bar = 100 µm. Transfection efficiency of muscle cells isolated from skeletal muscle of Japanese flounder and cultured with Dulbecco’s Modified Eagle Medium (DMEM) supplemented with 10% fetal bovine serum (FBS) at 23 °C for 24 h. Cells were treated with PBS (**a**,**d**) and transfected with pcDNA3.1-EGFP plasmid (**b**,**e**) and pcDNA3.1-MSTN-1-EGFP plasmid (**c**,**f**). Fluorescence image showed a certain number of EGFP-expressed cells of pcDNA3.1-EGFP group (28.99% ± 2.04%) (**e**) and pcDNA3.1-MSTN-1-EGFP group (28.62% ± 1.50%) in the fluorescein isothiocyanate (FITC) channel (**f**).

**Figure 6:**
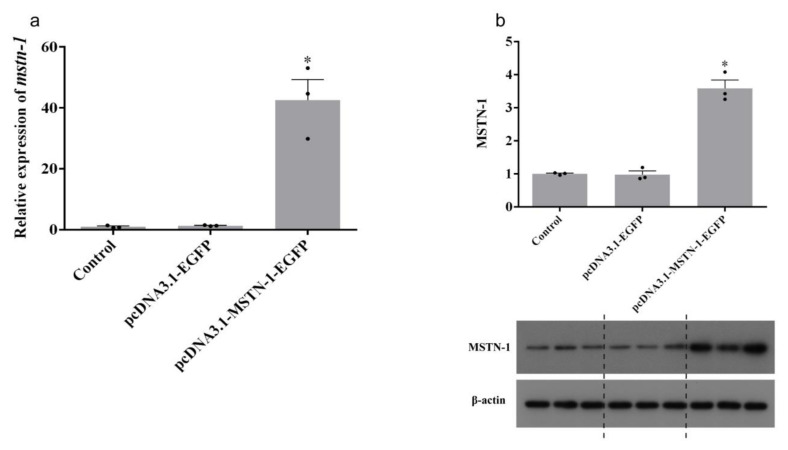
Relative expression levels of the mRNA (**a**) and protein (**b**) of MSTN-1 in primary cultured muscle cells treated with pcDNA3.1-MSTN-1-EGFP plasmid. Results are represented as mean ± SE (*n* = 3). * means significantly different compared with control (*p* < 0.05).

The transfection efficiency is shown in Figure 5. Compared with the control group, transfection with pcDNA3.1-EGFP plasmid did not influence the expression of *mstn-1* (*p* > 0.05), while the expression of *mstn-1* in pcDNA3.1-MSTN-1-EGFP plasmid group was significantly higher than that in the control group (*p* < 0.05) (Figure 6a). Similar result was also found in the protein expression of MSTN-1 (Figure 6b). *Myostatin-1* overexpression via pcDNA3.1-MSTN-1-EGFP transfection significantly increased the mRNA level and protein expression of MSTN-1 (*p* < 0.05).

### 3.6. Altered Proliferation of Primary Muscle Cells by mstn-1 Knockdown and Overexpression

**Figure 7:**
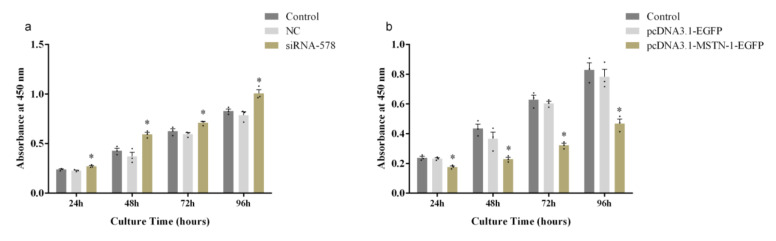
Change of cell proliferation by alternating the expression of *mstn-1*. (**a**) Absorbance at 450 nm of the cells from the control group, negative control (NC) group, and siRNA-578 group; (**b**) Absorbance at 450 nm of the cells from the control group, pcDNA3.1-EGFP group, and pcDNA3.1-MSTN-1-EGFP group. The absorbance was measured at 24, 48, 72, and 96 h after incubating in complete medium. * means significantly different compared with control at the same time point (*p* < 0.05).

To determine the effect of *mstn-1* knockdown and overexpression on the cell proliferation, primary muscle cells of Japanese flounder were transfected with siRNA-578 and pcDNA3.1-MSTN-1-EGFP, respectively; after that, the cells were cultured for 24, 48, 72, or 96 h in complete medium. The proliferation of cells was monitored using CCK-8 reagents. As shown in Figure 7a, the cells transfected with siRNA-578 had significantly higher density than the control group at all times (*p* < 0.05), while no significant difference was detected between the cells from NC group and cells from the control group (*p* > 0.05). The cells transfected with the pcDNA3.1-MSTN-1-EGFP had significantly lower density than the control group at all times (*p* < 0.05), while the transfection of pcDNA3.1-EGFP did not affect the cell density (Figure 7b). These results indicated that *mstn-1* negatively regulates cell proliferation.

### 3.7. Relative Expression of Muscle Growth and Proteolysis-Related Genes after Overexpression of mstn-1 in Muscle Cells of Japanese Flounder

**Figure 8:**
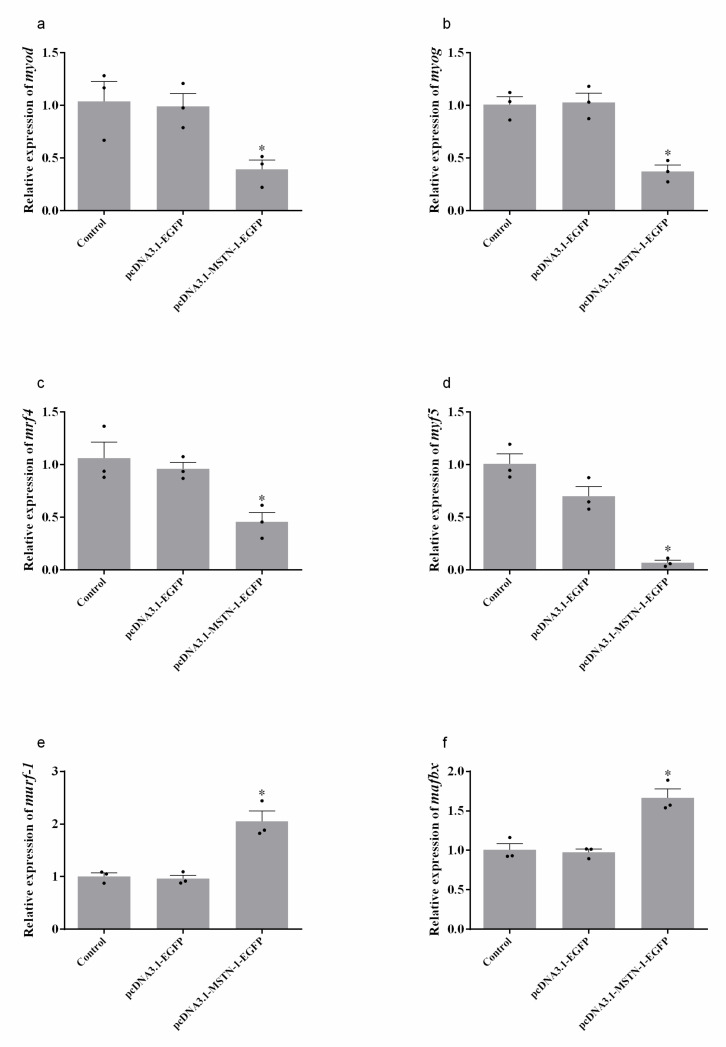
Relative expression of muscle growth-related genes and proteolysis-related genes after *mstn-1* overexpression in Japanese flounder primary muscle cells. (**a**) Relative expression of *myod*. (**b**) Relative expression of *myog*. (**c**) Relative expression of *mrf4*. (**d**) Relative expression of *myf5*. (**e**) Relative expression of *murf-1*. (**f**) Relative expression of *mafbx*. Results are represented as mean ± SE (*n* = 3). * means significantly different compared with control (*p* < 0.05).

Twenty-four hours after transfecting with pcDNA3.1-MSTN-1-EGFP, the mRNA levels of myogenic regulatory factors (MRFs) (*myod*, *myog*, *mrf4,* and *myf5*) were significantly decreased compared with the control group (*p* < 0.05). The expressions of proteolysis-related genes, muscle RING-finger protein 1 (*murf-1*) and muscle atrophy F-box protein (*mafbx*), were significantly increased compared with the control group (*p* < 0.05). The transfection of pcDNA3.1-EGFP did not influence the expression of these genes (*p* > 0.05). The data are shown in Figure 8.

### 3.8. Western Blot Analysis after the Overexpression of mstn-1 in Muscle Cells of Japanese Flounder

**Figure 9:**
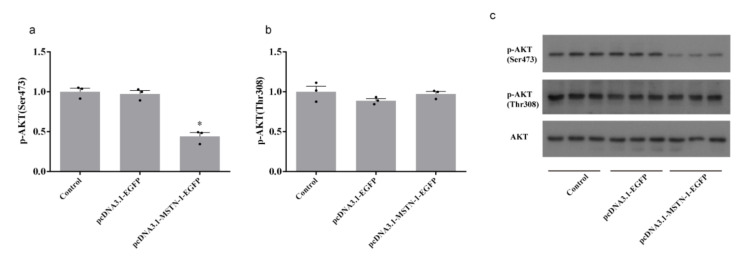
Overexpression of *mstn-1* inhibits protein kinase B (AKT) signaling by decreasing the phosphorylation of AKT at Ser473. (**a**) Western blot analysis of the phosphorylation level of AKT at Ser473. Histograms represent the ratio between the phosphorylated AKT at Ser473 and the total amount of AKT and (**b**) Western blot analysis of the phosphorylation level of AKT at Thr308. Histograms represent the ratio between the phosphorylated AKT at Thr308 and the total amount of AKT. (**c**) Effect of *mstn-1* overexpression on the protein expressions of phosphorylated protein kinase B (p-AKT) (Ser473), p-AKT (Thr308), and AKT. Results are represented as mean ± SE (*n* = 3). * means significantly different compared with control (*p* < 0.05).

**Figure 10:**
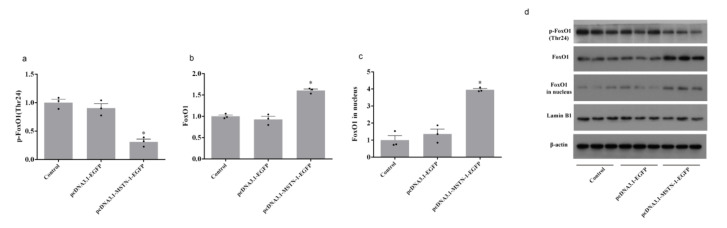
Overexpression of *mstn-1* decreases the phosphorylation of phosphor-Forkhead box O1 (FoxO1) as well as increases the nuclear accumulation of FoxO1. (**a**) Western blot analysis of the phosphorylation level of FoxO1. Histogram represents the ratio between the phosphorylated protein and the total amount of FoxO1. (**b**) Western blot analysis of FoxO1. Histogram represents the ratio between FoxO1 and β-actin. (**c**) Western blot analysis of FoxO1 in nucleus. Histograms represent the ratio between the FoxO1 in nucleus and Lamin B1 (as reference protein in nucleus). (**d**) Effect of *mstn-1* overexpression on the protein expression of p-FoxO1 (Thr24), FoxO1, and FoxO1 in nucleus. Results are represented as mean ± SE (*n* = 3). * means significantly different compared with control (*p* < 0.05).

**Figure 11:**
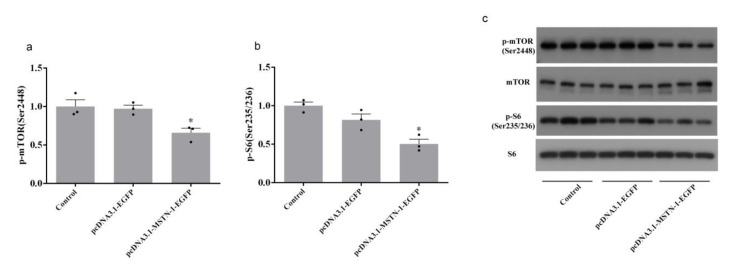
Overexpression of *mstn-1* inhibits the mammalian target of rapamycin (mTOR) signaling. (**a**) Western blot analysis of the phosphorylation level of mTOR. Histogram represents the ratio between the phosphorylated protein and the total amount of mTOR. (**b**) Western blot analysis of the phosphorylation level of S6. Histogram represents the ratio between the phosphorylated protein and the total amount of S6. (**c**) Effect of *mstn-1* overexpression on the protein expressions of p-mTOR (Ser2448), mTOR, p-S6 (Ser235/235), and S6. Results are represented as mean ± SE (*n* = 3). * means significantly different compared with control (*p* < 0.05).

Forty-eight hours after transfection with pcDNA3.1-MSTN-1-EGFP, the phosphorylation level of AKT at Ser473 was significantly decreased (*p* < 0.05), while the phosphorylation level of AKT at Thr308 was unchanged (*p* > 0.05) (Figure 9). The downregulation of phosphorylated FoxO1 (Thr24) (*p* < 0.05) and upregulation of FoxO1 (*p* < 0.05) was observed in cells transfected with pcDNA3.1-MSTN-1-EGFP. FoxO1 in nucleus was significantly increased by pcDNA3.1-MSTN-1-EGFP transfection (*p* < 0.05) (Figure 10). The phosphorylation levels of mTOR (Ser2448) and S6 (Ser235/236) were significantly decreased in the pcDNA3.1-MSTN-1-EGFP group (*p* < 0.05) (Figure 11).

## 4. Discussion

Evidence supports that MSTN is a strong negative regulator of muscle mass. It has been shown that MSTN can decrease the mRNA levels of *myod* and *myog* both in mammals and zebrafish [18,33]. The inhibition of mTOR signal pathway and promotion of ubiquitin-proteasomal degradative pathways by MSTN were demonstrated in both mammals and rainbow trout [29,34,35,36]. In rainbow trout, human recombinant MSTN induced the myotube atrophy [29]. The result showed the exogenous MSTN treatment can function in fish. In present study, the endogenous *mstn-1* was artificially regulated by siRNA knockdown and gene overexpression using an in vitro model to confirm the function and the underlying mechanisms of *mstn-1* in primary cultured muscle cells of Japanese flounder.

The present study showed that knockdown of *mstn-1* led to a significant elevated cell proliferation, while the *mstn-1* overexpression significantly inhibited cell proliferation. These results are consistent with the former study, in which C2C12 myoblasts with *MSTN* gene knockout showed a significant increase in proliferation [37]. The addition of human MSTN treatment had no effect on proliferation of trout myoblasts, while it decreased the proliferation of IGF1-stimulated myoblasts in a dose-dependent manner [16]. Combined with previous researches, the present results confirmed that the endogenous *mstn-1* had the growth inhibition effect in primary cultured Japanese flounder muscle cells.

Myogenic regulatory factors (MRFs), including MyoD, Myf5, MyoG, and Mrf4, regulate muscle hyperplasia and hypertrophy [38]. MyoD and Myf5 are primary MRFs that directly regulate proliferation of undifferentiated myoblasts, whereas MyoG and Mrf4 are considered secondary MRFs to control the differentiation and the fusion of myoblasts [38,39,40]. MSTN binds to type II serine/threonine kinase receptor to activate the Smad2/3 pathway and then suppresses the MRFs expression [41]. The present results showed that the knockdown of Japanese flounder *mstn-1* significantly increased the expression of MRFs (*myod*, *myf5*, *myog,* and *mrf4*), while the overexpression of Japanese flounder *mstn-1* resulted in significant downregulation of MRFs (*myod*, *myf5*, *myog,* and *mrf4*). Similar results were also found in medaka (*Oryzias latipes*). Chiang et al. (2015) mutated the genome sequence of *mstn* in medaka by genome editing with engineered nucleases [42], they found that in MSTN-/- F2 fish, the expression levels of *myod*, *myf5,* and *myog* were significantly increased. Knockdown of *mstn-1* by myostatin-1 morpholino injection in zebrafish embryos also led to the upregulation of muscle-specific transcription factors including *myod* and *myog* [18]. However, recombinant human MSTN treatment did not induce a decrease of *myod* or *myog* level in rainbow trout myoblasts [16]. Different results might be attributed to the different treatments and the experimental models used in different studies. Nevertheless, the present study showed a negative regulatory effect of endogenous *mstn-1* on MRFs expression in primary cultured muscle cells of Japanese flounder.

The mTOR/p70S6k signaling pathway is crucial to protein synthesis and cell growth. It is reported that p70S6K and 4E-BP1 are two regulatory proteins of protein synthesis [43,44]. Due to the shortage of appropriate antibodies, the phosphorylation level and total protein level of 4E-BP1 were not detected in this study. Nevertheless, the phosphorylation levels of mTOR and S6 were both significantly downregulated by the overexpression of *mstn-1* in Japanese flounder muscle cells. S6 is a primary substrate of p70S6K and its phosphorylation level reflects the phosphorylation level of p70S6K [45]. Previous researches demonstrated a high probability that the inhibitory effect of MSTN on mTOR signaling pathway is conserved between fish and mammalians [6,29]. Human MSTN was able to prevent the full activation of mTOR signaling by IGF1 in trout myotubes [29]. In the present study, the inhibited mTOR/p70S6K signaling pathway caused by overexpression of Japanese flounder *mstn-1* is similar to previous researches in other species [46,47]. Taken together, Japanese flounder *mstn-1* also plays an inhibitory function in protein synthesis by negatively regulating the mTOR/p70S6k signaling pathway.

Ubiquitin-proteasomal system (UPS) is an important proteolytic pathway involved in fish muscle atrophy [48]. Among the UPS members, MuRF-1 and MAFbx are key E3 ubiquitin ligases specifically expressed in muscle [49]. Elevated expression of MAFbx and MuRF-1 as well as enhanced protein degradation were found during MSTN-induced human myotube wasting [34]. FoxO transcription factor FoxO1 was reported to regulate atrophy-related genes and induce muscle atrophy [50,51]. MSTN was shown to activate FoxO1 and increase the expression of mafbx and murf-1 [35]. In the present study, the expression of *mafbx* and *murf-1* were induced by overexpression of *mstn-1*. Meanwhile, FoxO1 expression was upregulated and phosphorylated FoxO1 (inactive form) was reduced, which led to an accumulation of active FoxO1. The dephosphorylated FoxO1 plays its function by translocating to nucleus [52]. Thus, the nuclear protein was extracted and the FoxO1 in nucleus was detected in this study. The result showed that *mstn-1* overexpression led to a significant increase in FoxO1 in nucleus (Figure 10c). By this reason, the transcriptional activity of FoxO1 was supposed to be enhanced. As a consequence of enhanced FoxO1 function, the mRNA levels of *mafbx* and *murf-1* significantly increased in the pcDNA3.1-MSTN-1-EGFP group. A similar conclusion has been reached in an earlier study that human MSTN induced the expression of *mafbx* in rainbow trout myotubes and affected the phosphorylation of FoxO1 when treated by IGF1 [29]. On the contrary, myostatin gene deletion was shown to prevent glucocorticoid-induced muscle atrophy by suppressing the upregulation of mafbx and murf-1 [53]. In an earlier study performed on C2C12 cells, the IGF-1/PI3K/AKT hypertrophy pathway was confirmed to be reversed by MSTN and thereby the levels of active FoxO1 was increased [35]. To determine whether Japanese flounder *mstn-1* reduces the phosphorylated FoxO1 via the AKT pathway, levels of phosphorylated AKT were measured. The present results showed that Akt Ser473 phosphorylation was significantly decreased, whereas Akt Thr308 phosphorylation did not change with the overexpression of *mstn-1* in primary muscle cells. These results demonstrate that Ser473 is the critical phosphorylation site of Akt in response to *mstn-1* in Japanese flounder. The decreased phosphorylation levels of AKT and FoxO1 determines that *mstn-1* induces the expression of *mafbx* and *murf-1* via the AKT/FoxO1 signaling. However, the impact of MSTN on ubiquitin-proteasomal system (UPS) is still controversial. Some studies suggest that protein degradation was not regulated by MSTN [6,54]. In the present study, the UPS in primary cultured Japanese founder muscle cells was shown to be regulated by *mstn-1*. It is shown that the *mstn-1* plays an important role in the regulation of protein degradation in Japanese flounder.

## 5. Conclusions

**Figure 12:**
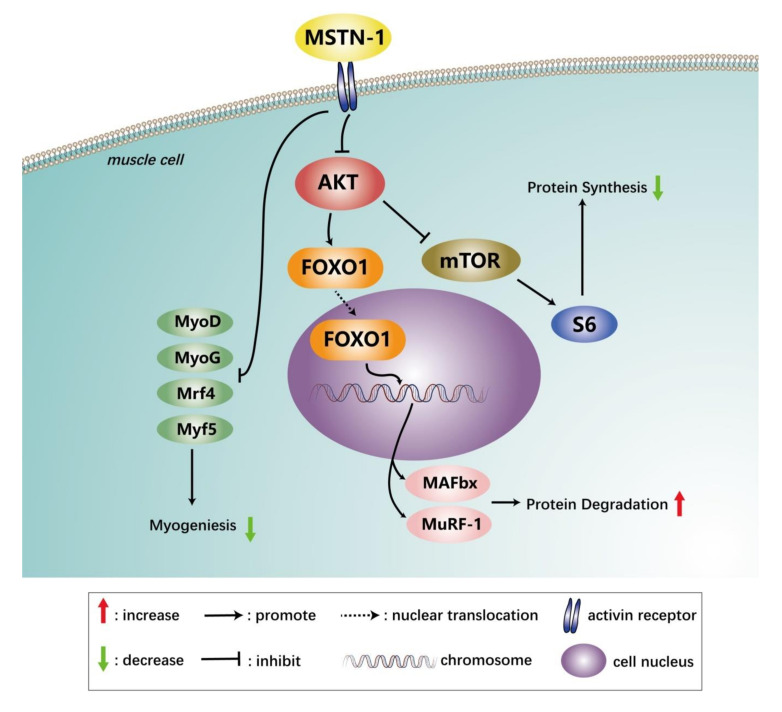
The proposed mechanism of myostatin-1 on muscle cell of Japanese flounder in the present study.

In conclusion, the present study found that *mstn-1* negatively regulates muscle cell proliferation and the mRNA expression of MRFs. The overexpression of *mstn-1* inhibited the activation of mTOR signal pathway and the phosphorylation of AKT at Ser 473. Meanwhile, it activated ubiquitin-proteasomal system via increasing dephosphorylation and nuclear localization of FoxO1 (Figure 12). These results demonstrate that the *mstn-1* has the effects of inhibiting cell proliferation and growth in muscle of Japanese flounder.

## Figures and Tables

**Table 1:** List of PCR primer pairs used for the real-time Q-PCR analysis.

Genes	Forward (5’-3’)	Reverse (5’-3’)	Accession No.
*myf5*	GCAACGCCATCCACTACATCG	TGCATTCAACTGGTGCCACACT	DQ872515
*myod*	GCAACGCCATCAGCTACATCG	CGTTTGGAGTCTGGGAGAAATAAG	DQ184914
*myog*	GTCTGGGGGTGTTGGAGTTGG	GACGCCTCTTCTCCCTCATCG	EF144128
*mrf4*	AGAGCAGCGGGGAGGAACAC	GACCTTGCAGGCCCACATGA	MK453386
*mstn-1*	TTTGAGGACTTTGGCTGGGACT	GCGACATCTTGGTGGGGGTA	DQ412048
*murf-1*	TTGTGCCGTAGTTGTGCTAGTGAC	CATGGCGATCAAGCACGACCTC	MK292717
*mafbx*	GCTGGGTGAAAACCGAGGAG	CTTCTTGGCAGCCATGTCGT	MK453387
*β-actin*	GGAAATCGTGCGTGACATTAAG	CCTCTGGACAACGGAACCTCT	HQ386788

**Table 2:** List of PCR primer pairs used for the reverse-transcriptase PCR (RT-PCR) analysis.

Genes	Forward (5’-3’)	Reverse (5’-3’)	Accession No.
*Pax7b*	AGCTAGCGGCATTCAACCAT	GTGTTGTGGCTGTGAGGAGA	KP323416
*myod*	GCAACGCCATCAGCTACATCG	CGTTTGGAGTCTGGGAGAAATAAG	DQ184914
*myog*	GAGTCTGTCTGGGGGTGTTG	ACTGCAGAGATGCTGTCCAC	EF144128
*mstn-1*	TTTGAGGACTTTGGCTGGGACT	GCGACATCTTGGTGGGGGTA	MK453386
*β-actin*	GAGCGTGGCTACTCCTTCAC	TACGCTCAGGTGGGGCAAT	HQ386788

## Supplementary Materials

The following are available online at https://www.mdpi.com/2073-4409/9/11/2376/s1, Table S1: List of siRNA used for *mstn-1* interfering.
