# Supplementary material for: Myostatin-1 Inhibits Cell Proliferation by Inhibiting the mTOR Signal Pathway and MRFs, and Activating the Ubiquitin-Proteasomal System in Skeletal Muscle Cells of Japanese Flounder Paralichthys olivaceus
Source: Cells. 2020 Oct 29;9(11):2376. doi: 10.3390/cells9112376 (PMC7692286; doi:10.3390/cells9112376)
Supplement: Supplementary file 1 [file cells-09-02376-s001.pdf]

cells

MDPI

Article

Myostatin-1 Inhibits Cell Proliferation by Inhibiting the mTOR Signal Pathway and MRFs, and Activating the Ubiquitin-Proteasomal System in Skeletal Muscle Cells of Japanese Flounder *Paralichthys olivaceus*

Jiahuan Liu, Mingzhu Pan, Dong Huang, Yanlin Guo, Mengxi Yang, Wenbing Zhang * and Kangsen Mai

The Key Laboratory of Aquaculture Nutrition and Feeds (Ministry of Agriculture and Rural Affairs); the Key Laboratory of Mariculture (Ministry of Education), Ocean University of China, Qingdao 266003, China; liujiahuan@stu.ouc.edu.cn (J.L.); pmz@stu.ouc.edu.cn (M.P.) huangdong@stu.ouc.edu.cn (D.H.); guoyanlin@stu.ouc.edu.cn (Y.G.); ymx@stu.ouc.edu.cn (M.Y.); wzhang@ouc.edu.cn (W. Z.); kmai@ouc.edu.cn (K.M.)

* Correspondence: wzhang@ouc.edu.cn; Tel.: +86-532-8203-2145

Table S1. List of siRNA used for *mstn-1* interfering.

siRNA	Sense (5′-3′)	Antisense (5′-3′)
siRNA-162	GCGACUAAACGCGAUCAUATT	UAUGAUCGCGUUUAGUCGCTT
siRNA-315	GGAUGUGGUCAUGGAGGAUTT	AUCCUCCAUGACCACAUCCTT
siRNA-578	CCCUGAAGAUUGACGUGAATT	UUCACGUCAAUCUUCAGGGTT
siRNA-677	GCAUCGAGAUUAACGCCUUTT	AAGGCGUUAAUCUCGAUGCTT
siRNA-912	GCGCUACAAGGCCAACUAUTT	AUAGUUGGCCUUGUAGCGCTT
siRNA-1070	GCAAAGAGCAGAUCAUCUATT	UAGAUGAUCUGCUCUUUGCTT
Si*mstn-1*-NC	UUCUCCGAACGUGUCACGUTT	ACGUGACACGUUCGGAGAATT
